# PD58 The Repellent Effects Of *Cymbopogon Nardus* On *Aedes Aegypti* Mosquitoes: A Rapid Review

**DOI:** 10.1017/S0266462324003167

**Published:** 2025-01-07

**Authors:** Ana Célia Rocha Medeiros, Allancleriston Alves Galdino, Edmar Candeia Gurjão, Erika Barbosa Camargo

## Abstract

**Introduction:**

Arboviruses transmitted by the *A. aegypti* mosquito are a public health problem in Brazil. Citronella, known for its repellent properties, has been suggested as a possible sustainable and natural preventive measure against the arbovirus triad. This study aimed to carry out a rapid review of the efficacy and safety of the repellent properties of citronella for the *A. aegypti* mosquito.

**Methods:**

The rapid review followed methods proposed by the Joanna Briggs Institute. Searches were conducted in the following literature databases: PubMed, LILACS, Embase, Scopus, Web of Science, and the Cochrane Library. Quality assessment was carried out using the AMSTAR-2 tool. The review aimed to determine the efficacy and safety of citronella (*C. nardus*) as a repellent for the *A. aegypti* mosquito, compared with usual methods.

**Results:**

Citronella repels *A. aegypti* mosquitoes for between 12 and 480 minutes, depending on the concentration and formulation of the product. Considering its protection time, reapplying the product doubles the protective effect. Adding vanillin to the formula reduces the product’s volatility. Citronella is not absorbed through the skin like DEET products, making it less toxic.

**Conclusions:**

Citronella-based products can be used as a complementary measure of protection against arboviruses. Additional investigations are needed on the percentage of essential oil present in homemade formulations. Studies addressing the safety of citronella are imperative for its use in the public health system. Controlled studies evaluating its degree of repellency are also needed.

